# Coadministration of Stigmasterol and Dexamethasone (STIG+DEX) Modulates Steroid-Resistant Asthma

**DOI:** 10.1155/2022/2222270

**Published:** 2022-08-24

**Authors:** Abigail Hohoayi, Aaron O. Antwi, Veronica Amoah, Newman Osafo, Paul P. O. Sampene, George Ainooson

**Affiliations:** ^1^Department of Pharmacology and Toxicology, School of Pharmacy, University of Health and Allied Sciences (UHAS), Ho, Ghana; ^2^Department of Pharmacology, Faculty of Pharmacy and Pharmaceutical Sciences, College of Health Sciences, Kwame Nkrumah University of Science and Technology (KNUST), Kumasi, Ghana; ^3^Department of Pathology, School of Medicine and Dentistry, Kwame Nkrumah University of Science and Technology (KNUST), Kumasi, Ghana

## Abstract

Airway inflammation in asthma is managed with anti-inflammatory steroids such as dexamethasone (DEX). However, about 20% of asthmatics do not respond to this therapy and are classified as steroid-resistant. Currently, no effective therapy is available for steroid-resistant asthma. This work therefore evaluated the effect of a plant sterol, stigmasterol (STIG), and stigmasterol-dexamethasone combination (STIG+DEX) in LPS-ovalbumin-induced steroid-resistant asthma in Guinea pigs. To do this, the effect of drugs on inflammatory features such as airway hyperreactivity and histopathology of lung tissue was evaluated. Additionally, the possible pathway of drug action was assessed by measuring events such neutrophil levels, oxidative and nitrative stress, and histone deacetylase 2 (HDAC2) and interleukin 17 (IL-17) levels. STIG alone did not affect inflammatory features, although it caused some changes in the molecular events associated with steroid-resistant asthma. However, STIG+DEX caused significant modulation of inflammatory features by protecting against destruction of lung tissue. The modulation of inflammatory features was associated with significant inhibition of neutrophilia and oxidative and nitrative stress, decrease in HDAC2, and increase in IL-17 levels that are usually associated with steroid-resistant asthma. Our findings show that although STIG and DEX individually do not protect against steroid-resistant asthma, their coadministration results in significant modulation of inflammatory features and the associated molecular events that lead to steroid-resistant asthma.

## 1. Introduction

Anti-inflammatory steroids such as glucocorticoids are highly effective in the treatment of inflammation and are currently recommended for management of persistent asthma as well as the preferred therapy for control of airway inflammation [1, 2]. However, a subset of asthmatics do not respond to steroid therapy and are therefore at risk of worsening disease severity [3, 4]. Not only that, steroid-resistant asthmatics visit the hospitals more frequently and are given high doses of anti-inflammatory steroids in attempt to manage the disease [5]. Associated with high intake of anti-inflammatory steroids or glucocorticoids are serious side effects such as osteoporosis, adrenal insufficiency, Cushing syndrome, and obesity [3, 5]. These side effects adversely affect the patient's general, social, and economic well-being [4].

Studies have shown that this class of asthmatics clinically show predominantly high levels of lipopolysaccharide (LPS) in their airway and sputum [6–8]. LPS, an endotoxin from Gram-negative bacteria, ever-present in the environment, is known to be one of the environmental causes of steroid resistance in asthma. Mechanistically, LPS is recognised by pattern recognition receptors such as the toll-like receptors (TLRs), CD14, and collectins, which include pulmonary surfactant proteins. TLR activation triggers a signalling cascade leading to the activation and nuclear translocation of nuclear factor kB, resulting in a proinflammatory cytokine response including neutrophils and macrophages influx. Subsequent doses or presence of LPS as seen in this study then evoke an increased release of bronchoconstrictive substances such as NO by iNOS, neutrophil, and macrophage-derived free radicals [9–11]. This pool of compounds released into the airway and the presence of an allergen (ovalbumin) cause inflammation of the airways, airway hyperreactivity, narrowed and mucus-filled bronchioles, coughing, wheezing, and difficulty in breathing [8, 11]. Mechanistically, there is the degradation of key enzymes such as histone deacetylase 2 (HDAC2). The degradation of HDAC2 inhibits anti-inflammatory gene transcription but activates the transcription of proinflammatory genes such as IL-17 thereby enhancing airway inflammation as well as the severity of the disease [12–14].

The current therapy for steroid-resistant asthma involves the use of glucocorticoids such as dexamethasone which is largely not effective [2, 4]. This therefore calls for intensive search for alternative therapeutic agents for the management of the disease. Therefore, this study investigated stigmasterol (STIG), a plant sterol with potent anti-inflammatory and antioxidant properties [15], as a potential drug for the management of steroid-resistant asthma using the LPS-ovalbumin-induced steroid-resistant asthma model in Guinea pigs. This model vividly portrays the molecular events and pathophysiology such as infiltration of immune cells (eosinophils and neutrophils) and inflammatory mediators, reduction in levels of HDAC2, and increased cytokine IL-17 expression associated with steroid-resistant asthma [6–8].

## 2. Materials and Methods

### 2.1. Materials

Stigmasterol (STIG) (95%), dexamethasone (DEX), polyethylene glycol (PEG), ovalbumin (OVA), lipopolysaccharide (LPS), and aluminium hydroxide, (Al(OH)_3_) were acquired from Sigma-Aldrich (St. Louis, USA). IL-17, HDAC2, and inducible nitric oxide synthase (iNOS) Enzyme-Linked Immunosorbent Assay (ELISA) quantification kits were purchased from Biobase China.

#### 2.1.1. Animals

Guinea pigs (American Guinea pigs) of both sexes were acquired from the animal house facility of the Department of Pharmacology, KNUST (Kumasi, Ghana). The animals were kept in standard environmental conditions of temperature, relative humidity, and normal dark and light cycles in the animal house facility and had access to commercial food and clean water without restrictions. All protocols used in this study were approved by the Faculty of Pharmacy and Pharmaceutical Sciences Ethics Committee, and animal handling was done in compliance with animal handling in accordance with regulations for animal welfare (USDA 1985; US Code, 42 USC § 289d) and the Public Health Service Policy on Humane Care and Use of Laboratory Animals (PHS, 2002).

### 2.2. Methods

#### 2.2.1. LPS-OVA Induced Steroid-Resistant Asthma in Guinea Pigs

**Figure 1:**
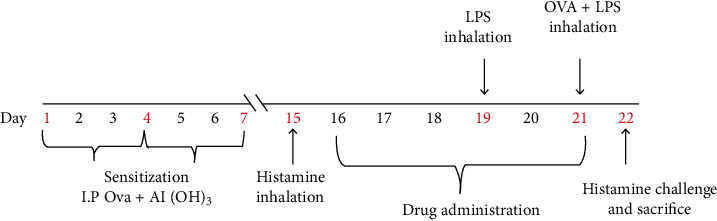
A schematic representation of the LPS-OVA-induced steroid-resistant asthma in Guinea pigs.

Sensitization and histamine inhalation, OVA-LPS challenge, drug administration, and sacrifice of animals as performed in this work are described in Figure 1.

*(1) Sensitization and Histamine Inhalation*. Guinea pigs were put into 6 groups, *n* = 5. Group 1 (vehicle control group) was sham sensitized by intraperitoneal (i.p) injection with normal saline (vehicle) only on days 1, 4, and 7, while groups 2 to 6 were sensitized by i.p injection of OVA (150 mg) and Al(OH)_3_ (100 mg) in saline (1 ml) on days 1, 4, and 7. On day 15, sham-sensitized Guinea pigs in group 1 were challenged with normal saline inhalations in a Perspex chamber using a nebulizer for 10 minutes while Guinea pigs in groups 2 to 6 were challenged with 2% histamine in normal saline inhalation in a Perspex chamber using a nebulizer for 10 minutes.

*(2) Drug Administration*. On days 16 to 21, Guinea pigs were given the following drug treatments:

- Group 1 received 1 ml i.p injection of normal saline (vehicle control)
- Group 2 received 1 ml i.p injection of 50% PEG (disease control)
- Group 3 was administered 1 ml i.p injection of 3 mg/kg dexamethasone in 50% PEG (DEX 3 mg/kg)
- Group 4 received 1 ml i.p injection of 50 mg/kg stigmasterol in 50% PEG (STIG 50 mg/kg)
- Group 5 received 1 ml i.p injection of 100 mg/kg stigmasterol in 50% PEG. (STIG 100 mg/kg)
- Group 6 received 1 ml i.p injection of a formulation of 100 mg/kg stigmasterol and 3 mg/kg dexamethasone in 50% PEG (STIG+DEX)

*(3) Lipopolysaccharide (LPS) Inhalation*. On day 19, six hours after drug administration, normal saline inhalation challenge was performed on Guinea pigs in group 1 while LPS (30 *μ*g/ml) inhalation challenge was performed on Guinea pigs in groups 2 to 6 in a Perspex chamber using a nebulizer for 10 minutes.

*(4) Ovalbumin and Lipopolysaccharide (LPS) Inhalation*. On day 21, six hours after drug treatment, Guinea pigs in group 1 were exposed to normal saline only while groups 2 to 6 were exposed to a coadministration of LPS (30 *μ*g/ml) and OVA (300 *μ*g/ml) in a Perspex chamber using a nebulizer for 10 minutes.

*(5) Sacrifice*. Six hours after histamine challenge on day 22, Guinea pigs were sacrificed with sodium pentobarbitone. Bronchoalveolar lavage (BAL) fluid of the whole lung was aspirated twice with normal saline (1 ml/100 g of Guinea pig weight) instilled through a polypropylene cannula, inserted into the trachea, and left for 3 min. BAL fluid was pooled and centrifuged at 3140*g* for 15 min.

#### 2.2.2. Analysis of Inflammatory Cell Infiltration: Neutrophil, Eosinophil, and Protein Concentration Analysis

Leukocyte subpopulations of eosinophils and neutrophils were quantified from BAL fluid using an automated analyser. Protein concentration was determined in the collected supernatant using the Bradford method [16].

#### 2.2.3. Analysis of Oxidative Stress Markers

*(1) Myeloperoxidase (MPO)*. MPO content was determined by using a modified *o*-dianisidine method. The assay contained 0.3 ml of 0.05 M phosphate buffer (pH 6.0), 0.3 ml of 0.01 M H_2_O_2_, and 0.5 ml of 0.02 M *o*-dianisidine (freshly prepared) in deionized water and 10 *μ*l supernatant in a final volume of 3.0 ml. The BAL fluid supernatant was added last, and the change in absorbance at 460 nm was read for 10 min (absorbance taken every 1 min) using a Synergy H1 Hybrid Reader spectrophotometer (BioTek Technologies, Winooski, VT, USA). Measurements were done in triplicates. The enzyme activity was extrapolated from standard peroxidase activity curve with the equation *y* = 2.0843*x* + 0.8672, where *y* is the absorbance at 460 nm and *x* is the enzyme activity. One unit of MPO is well defined, giving a rise in absorbance of 0.001 min^−1^. Specific activity was stated as U/mg protein.

*(2) Malondialdehyde (MDA)*. Using Heath and Packer method [17] and with minor modifications, MDA levels were measured. Briefly, 0.5 ml BAL fluid was added to 1.5 ml mixture of trichloroacetic acid (TCA) (20%) and thiobarbituric acid (TBA) (0.5%). The mixture was heated at 95°C for 30 min, immediately cooled and centrifuged at 15700*g* for 10 min. Further, 200 *μ*l volume of the supernatant was pipetted into a 96-well plate and the absorbance was read at 532 nm and 600 nm using a Synergy H1 Hybrid Reader spectrophotometer (BioTek Technology, VT, USA). With the equation below, MDA concentration was calculated with a molar extinction coefficient of 1.56 × 10^−5^ M^−1^ cm^−1^.

(1) $$\begin{array}{c} \text{nmol MDA per mg protein}=\left[\frac{\text{Absorbance }532\text{ nm}–\text{Absorbance }600\text{ nm}}{1.56\times 10^{-5}\times \text{total protein}}\right]\times 10^{6}. \end{array}$$

*(3) Catalase (CAT)*. The catalase assay as described by Sinha [18] was used with slight modifications. A 100*μ*l aliquot of BAL fluid, 1ml phosphate buffer (0.01M, pH7.0), and 400*μ*l H_2_O_2_ (1.18M) were added, and the mixture was incubated at room temperature for 5min. The reaction was halted by adding 2ml of a 3 : 1 mixture of glacial acetic acid and dichromate (5%). Absorbance was measured at 620nm with a Synergy H1 Hybrid Reader spectrophotometer (BioTek Technologies, Winooski, VT, USA). One unit of catalase activity, defined as the amount of enzymes that degrades 1mmol H_2_O_2_ per min at 25°C and pH7.0, was expressed in terms of its molar extinction coefficient of 39.4M^−1^cm^−1^ with the equation

(2) $$\begin{array}{c} \text{mUnitCAT}/\text{mg protein}=\left[\frac{\text{Absorbance at }620\text{ nm}}{3.94\times \text{weight of protein}}\right]\times 1000. \end{array}$$

*(4) Superoxide Dismutase (SOD)*. Misra [19] SOD activity assay was used in the estimation of SOD levels. Briefly, 500 *μ*l BAL fluid was treated with 150 *μ*l ice-cold chloroform and 750 *μ*l ethanol (96% *v*/*v*), vortexed for 1 min and then centrifuged at 2000 rpm for 20 min. 500 *μ*l portion of the supernatant, 500 *μ*l EDTA (0.6 mM), and 1 ml carbonate bicarbonate buffer (0.1 M, pH 10.2) were added. The reaction was initiated by the addition of 50 *μ*l adrenaline (1.3 mM). Absorbance was measured with a Synergy H1 Hybrid Reader spectrophotometer (BioTek Technologies, Winooski, VT, USA) at 480 nm against a blank. Activity of SOD, measured as the quantity of the enzyme required to inhibit the autooxidation of adrenaline, was calculated using the equation

(3) $$\begin{array}{c} \%\text{inhibition}=\left[\frac{\text{Absorbance }\left(\text{test}\right)-\text{Absorbance}\left(\text{blank}\right)}{\text{Absorbance}\left(\text{test}\right)}\right]\times 100. \end{array}$$

SOD level was expressed in units per mg protein, where 1 unit of enzyme activity is the quantity of enzyme required to prevent the autooxidation of adrenaline at 25°C, and calculated with the equation

(4) $$\begin{array}{c} \text{Units of SOD activity}/\text{mg protein}=\left[\frac{\%\text{inhibition}}{50\times \text{weight of protein}}\right]\times 100. \end{array}$$

*(5) Reduced Glutathione (GSH)*. GSH levels were determined by a method earlier described by Ellman [20]. Briefly, 100 *μ*l BALF aliquot was mixed with 2.4 ml EDTA (0.02 M) at 4°C for 10 min. 2 ml distilled water and 500 *μ*l TCA 50% were added and centrifuged at 3000 rpm for 5 min. 1 ml of the supernatant, 50 *μ*l 5,5′-dithiobis-2-nitrobenzoic acid (DTNB) (10 mM), and 2 ml Tris buffer (0.4 M, pH 8.9) were added. The absorbance was read within 5 min of DTNB addition at 412 nm against a blank (reagents only) with a Synergy H1 Hybrid Reader spectrophotometer (BioTek Technologies, Winooski, VT, USA). The final sulfhydryl concentration was extrapolated from a standard curve with the equation *y* = 0.8916*x* − 0.0477 where *x* is the absorbance at 412 nm.

*(6) Nitric Oxide (NO)*. Nitric oxide concentration was measured in BAL fluid using a Griess assay method. Briefly, 100 *μ*l aliquots of BAL fluid was incubated with 100 *μ*l Griess reagent (1% sulfanilamide, 0.05% naphthyl ethylenediamine dihydrochloride, 2.5% H_3_PO_4_) at room temperature for 10 min. The absorbance was read with a Synergy H1 Hybrid Reader spectrophotometer (BioTek Technologies, Winooski, VT, USA) at 550 nm using sodium nitrite as a standard and double-distilled water as blank. BAL fluid free medium contained 0.2 to 0.3 nmol of NO_2_^−^ per well. The absorbance was determined for each experiment and subtracted from the value obtained for BAL fluid experiment. A standard curve of the absorbance to concentration obtained from the BAL fluid free mediums (0.2 to 0.3 nmol NO_2_^−^) was drawn and the values of the concentration of NO_2_^−^ in the BAL fluid medium extrapolated from the standard curve with the formula *y* = 2.8864*x* − 0.4023.

#### 2.2.4. ELISA of IL-17, HDAC2, and iNOS

The levels of IL-17, HDAC2, and iNOS were measured using ELISA and according to manufacturer's protocol for each assay. To do this, 1 mg lung tissue was homogenized on ice using phosphate buffer, the homogenate was centrifuged at 6280*g* for 20 min, and the supernatant was used for the assay.

#### 2.2.5. Histology of Lung Tissues

Lung tissues were carefully removed and fixed in 10% formalin. Tissues were serially dehydrated in increasing concentrations of ethanol, cleared in xylene in a TP 1020 tissue processor (Leica Biosystems, Wetzlar, Germany), and embedded in paraffin using a Leica EG 1160 embedding machine (Leica Biosystems, Wetzlar, Germany). Transverse sections of 3 *μ*m were cut with a Leica RM 2125 Microtome (Leica Biosystems, Wetzlar, Germany), deparaffinized, and hydrated with distilled water. Tissue sections were stained with H and E appropriately for airway integrity examination and observed under light microscope for assessment of smooth muscle hyperplasia of the airway.

Scoring was done according to Gibson–Corley [21]. Scoring adopted was 0—normal smooth muscle hyperplasia, 1—mild smooth muscle hyperplasia, 2—moderate smooth muscle hyperplasia, and 3—severe smooth muscle hyperplasia.

#### 2.2.6. Statistical Analysis

Data are presented as mean ± standard error mean (SEM). The analysis of data was performed with one-way analysis of variance (ANOVA). Multiple comparisons between the treatment groups were performed using Dunnett's *post hoc test*. All statistical analyses were done using GraphPad for Windows version 6 (GraphPad Prism Software, San Diego, USA).

## 3. Results

### 3.1. STIG and STIG+DEX Protect against Inflammation

#### 3.1.1. STIG+DEX Inhibits Rise in Neutrophil and Eosinophil Levels in BAL Fluid

Increased levels of immune cells such as neutrophils and eosinophils have been implicated in steroid-resistant asthma with increased neutrophil infiltration over eosinophils [22].

**Figure 2:**
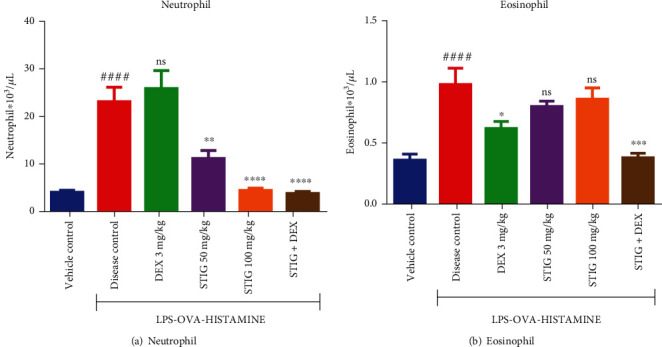
Effects of STIG and STIG+DEX on neutrophil and eosinophil levels. Data is expressed as mean ± SEM. ^####^*p* < 0.0001 compared to the vehicle control group; ^∗∗∗∗^*p* < 0.0001, ^∗∗∗^*p* < 0.001, ^∗∗^*p* < 0.01, ^∗^*p* < 0.05, and ns (not significant) as compared to the disease control group using one-way ANOVA followed by Dunnett's *post hoc test.*

The disease control in this study showed significant levels of neutrophils compared to eosinophils. The neutrophil level in the disease control was 4-fold increase (*p* < 0.0001) at 23.20 ± 3.12 × 10^3^/*μ*l (Figure 2(a)), compared to the vehicle control which had a lower neutrophil level of 4.00 ± 0.45 × 10^3^/*μ*l. This high neutrophil level shown by the disease condition was not reduced by DEX. Nevertheless, the STIG at both doses (50 and 100 mg/kg) showed lower neutrophil levels (11.2 ± 1.7 × 10^3^/*μ*l and 4.4 ± 0.5 × 10^3^/*μ*l, respectively) representing a 51.72 ± 1.42% (*p* < 0.01) and 81.03 ± 2.62% (*p* < 0.0001) decrease, respectively. Even more, STG+DEX decreased neutrophil levels significantly by 83.62 ± 2.75% (*p* < 0.0001) to 3.80 ± 0.37 × 10^3^/*μ*l. Although neutrophils are central mediators of steroid-resistant asthma, eosinophils also play a role.

The effect of the drugs on eosinophil levels in the BAL fluid is as follows: the disease control recorded a 3-fold increase (*p* < 0.0001) in eosinophil levels at 0.93 ± 0.05 × 10^3^/*μ*l (Figure 2(b)), against 0.36 ± 0.05 × 10^3^/*μ*l in the vehicle control. Though DEX reduced eosinophil levels by 33.33 ± 0.01% at 0.62 ± 0.06 × 10^3^/*μ*l (*p* < 0.1), interestingly STIG 50 mg/kg and STIG 100 mg/kg did not cause any substantial reduction in the eosinophil levels. However, STIG+DEX significantly reduced the eosinophil levels appreciably by 59.14 ± 0.01% (*p* < 0.001) at 0.38 ± 0.04 × 10^3^/*μ*l. The immense reduction in immune cell levels by STIG+DEX is a crucial indication that reducing levels of immune cells plays a significant role in managing steroid-resistant asthma.

#### 3.1.2. STIG and STIG+DEX Mitigate Rise in MPO and MDA Levels

Influx of immune cells into the airway releases oxidative stress agents which destroys lipid membranes and induces airway inflammation [4].

**Figure 3:**
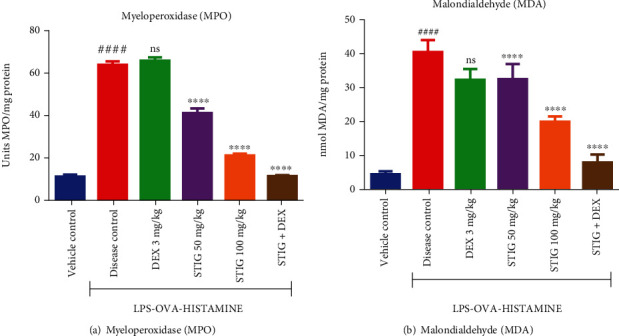
Effect of STIG and STIG+DEX on MDA and MPO levels. Data is expressed as mean ± SEM. ^####^*p* < 0.0001 compared to the vehicle control group; ^∗∗∗∗^*p* < 0.0001 and ns (not significant) as compared to the disease control group using one-way ANOVA followed by Dunnett's *post hoc test.*

The level of a direct lipid peroxidation product, MDA, increased significantly by 7-fold recording 40.56 ± 3.54 nmol/mg protein (*p* < 0.0001) in the disease control relative to levels of 4.55 ± 0.88 nmol/mg protein in the vehicle control (Figure 3(a)). This evident rise in MDA levels was not inhibited by DEX, but STIG at both doses (50 and 100 mg/kg) significantly reduced MDA levels (32.55 ± 4.46 nmol/mg protein and 20 ± 1.52 nmol/mg protein, respectively) representing 19.75 ± 0.92% (*p* < 0.0001) and 50.69 ± 2.02% (*p* < 0.0001) decrease, respectively. More so, STIG+DEX decreased MDA levels significantly by 80.55 ± 1.18% (*p* < 0.0001) at 7.89 ± 2.36 nmol/mg protein.

Also, levels of myeloperoxidase (MPO) increased by 4-folds (*p* < 0.0001) at 63.93 ± 1.60 U/mg protein in the disease control compared to 11.17 ± 0.96 U/mg protein in the vehicle control (Figure 3(b)). DEX caused no substantial decrease in the MPO levels whereas STIG at 50 and 100 mg/kg caused significant 35.66 ± 0.58% (*p* < 0.0001) and 67.02 ± 0.70% (*p* < 0.0001) reductions in MPO levels, respectively, recording 41.13 ± 2.18 U/mg protein and 21.08 ± 0.90 U/mg protein, respectively. STIG+DEX was more effective as it caused the highest reduction in MPO level at 11.39 ± 0.45 U/mg protein, an appreciable 82.18 ± 1.15% (*p* < 0.0001) reduction. The reduction in levels of MPO as well as MDA by STIG and STIG+DEX is a desirable influence in managing airway inflammation in steroid-resistant asthma.

#### 3.1.3. STIG and STIG+DEX Inhibit Decrease in Superoxide Dismutase (SOD), Catalase (CAT), and Glutathione (GSH) Levels

**Figure 4:**
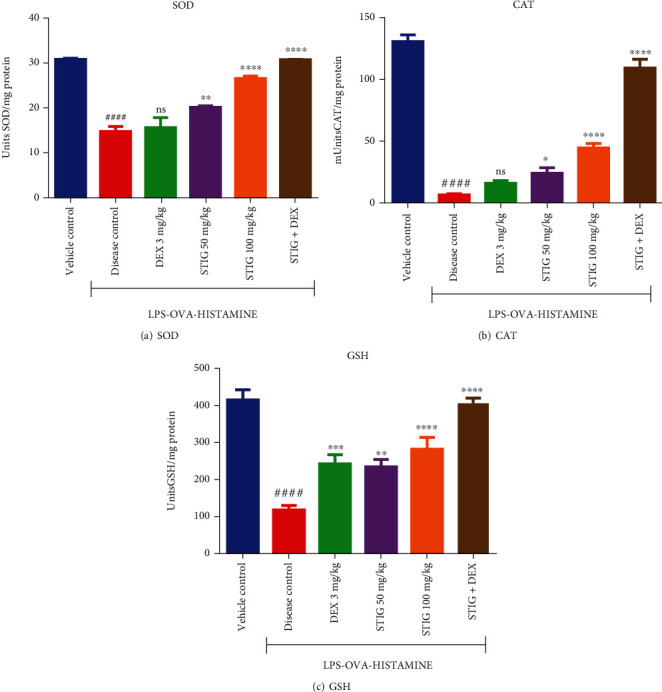
Effect of STIG and STIG+DEX on SOD, CAT, and GSH levels. Data is expressed as mean ± SEM. ^####^*p* < 0.0001 compared to the vehicle control group; ^∗∗∗∗^*p* < 0.0001, ^∗∗∗^*p* < 0.001^∗∗^*p* < 0.01, ∗*p* < 0.05, and ns (not significant) as compared to the disease control group using one-way ANOVA followed by Dunnett's *post hoc test*.

Downregulation of key antioxidant enzymes (SOD, CAT) and the molecule GSH is implicated in the exacerbation of inflammatory response, which occurs in steroid-resistant asthma [23]. In this work, as expected, the levels of the aforementioned agents (SOD, CAT, and GSH) were significantly lower by at least 2-fold (*p* < 0.0001) in the disease control group when compared to the vehicle control group (Figure 4). However, the ability of the drug treatment to reverse these effects was variable.

Concerning SOD (Figure 4(a)), the anti-inflammatory drug, DEX, did not cause any significant increase in SOD levels, but the natural plant sterol STIG at 50 and 100 mg/kg showed higher SOD levels (20.21 ± 0.34 and 26.66 ± 0.52 units/mg protein, respectively) representing 36.02 ± 0.78% (*p* < 0.01) and 79.52 ± 0.60% (*p* < 0.0001) increase in SOD levels, respectively, when compared to the disease control. STIG+DEX recorded even higher levels of about 30.82 ± 0.08 units/mg protein which is 107.54 ± 1.04% (*p* < 0.0001) increase in SOD levels when compared to the disease control group.

This trend was similar for CAT (Figure 4(b)); DEX did not cause any significant rise in CAT levels. But STIG at all the doses tested (50 and 100 mg/kg) increased CAT levels to 24.15 ± 4.10 and 44.57 ± 3.29 mUnits/mg protein, representing 253.96 ± 3.61% (*p* < 0.1) and 553.51 ± 2.80% (*p* < 0.0001) increase, respectively. Even more, STIG+DEX effect was 109.0 ± 6.66 mUnits/mg protein, which is a noteworthy 1498.24 ± 6.17% (*p* < 0.0001) increase in CAT levels when compared to the disease control.

On the other hand, GSH levels (Figure 4(c)) were elevated by DEX treatment to 244.20 ± 24.62 units/mg which is a substantial 105.04 ± 12.85% (*p* < 0.001) increase compared to the disease control. STIG (50 and 100 ) mg/kg also increased GSH levels to 236.2 ± 20.02 and 284.10 ± 31.83 units/mg protein causing 98.32 ± 8.25% (*p* < 0.01) and 138.54 ± 20.06% (*p* < 0.0001) increase, respectively. STIG+DEX caused the highest percentage increase in GSH levels (242.91 ± 2.52%, *p* < 0.0001) at 408.40 ± 14.29 units/mg protein.

This consistent increase in SOD, CAT, and GSH by notably STIG and STIG+DEX correlates well with their protective effects against asthma-induced dyspnea in Guinea pigs, suggesting that enhancement of antioxidant capacity by the treatment may have contributed to the ameliorative effect of STIG+DEX in the current model of steroid-resistant asthma which is desirable and crucial in managing airway inflammation in steroid asthma.

#### 3.1.4. STIG and STIG+DEX Inhibit Elevation in Inducible Nitric Oxide Synthase (iNOS) and Nitric Oxide (NO) Levels

**Figure 5:**
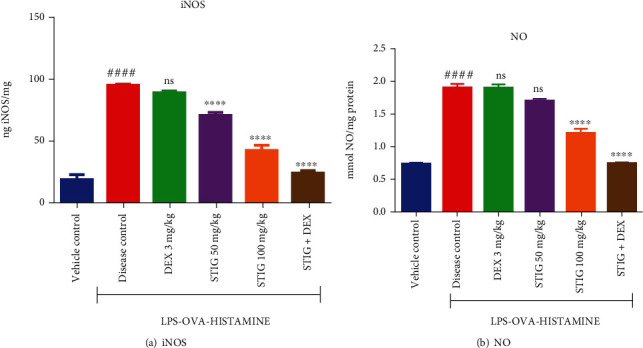
Effect of STIG and STIG+DEX on nitric oxide and inducible nitric oxide synthase levels. ^####^*p* < 0.0001 compared to the vehicle control group; ^∗∗∗∗^*p* < 0.0001 and ns (not significant) as compared to the disease control group by one-way ANOVA and then by Dunnett's *post hoc test.*

Increased levels of the prooxidation agents iNOS and NO have also been implicated in steroid-resistant asthma [24]. Not surprisingly, the LPS-OVA-induced asthma response as seen in the disease control resulted in increased levels of iNOS and NO (Figures 5(a) and 5(b)) resulting in 4- and 2-fold elevation (*p* < 0.0001), respectively, when compared to the disease control. DEX did not cause any significant reduction in the levels of both iNOS and NO, but STIG at all the doses tested (50 and 100 mg/kg) showed higher iNOS levels (70.89 ± 2.56 ng/mg and 32.86 ± 0.33 ng/mg protein), respectively, representing 25.78 ± 1.55% (*p* < 0.0001) and 65.58 ± 0.6% (*p* < 0.0001) decrease in iNOS level, respectively, when compared to disease control. STIG+DEX as previously observed was more effective, as it caused a 74.62 ± 0.92% (*p* < 0.0001) reduction in iNOS levels at 24.23 ± 1.93 ng/mg protein.

The pattern for NO modulation by drugs was not exactly as observed for iNOS. In this case, STIG at 50 mg/kg did not have any significant effect on NO level, but the 100 mg/kg STIG, induced a 36.4 ± 0.01% (*p* < 0.0001) decrease compared to disease control, as it decreased NO levels to 1.2 ± 0.07  mmol/mg protein. STIG+DEX treatment as observed earlier was the most effective treatment, as it caused a 61.05 ± 0.04% (*p* < 0.0001) reduction in NO levels, recording 0.74 ± 0.02 mmol/mg protein. This further confirms that enhancement of antioxidant capacity by STIG+DEX treatment may be important for its ameliorative effect in steroid-resistant asthma.

### 3.2. STIG+DEX Protects the Airway in Steroid-Resistant Asthma

#### 3.2.1. STIG Inhibits Decrease in HDAC2 While STIG+DEX Inhibits Decrease in HDAC2 as well as Increase in IL-17 Levels

Interconnected with influx of inflammatory mediators and increase in immune cells as it occurs in steroid insensitive asthma are the depletion of the enzyme HDAC2 and elevation of the proinflammatory cytokine IL-17 [24].

**Figure 6:**
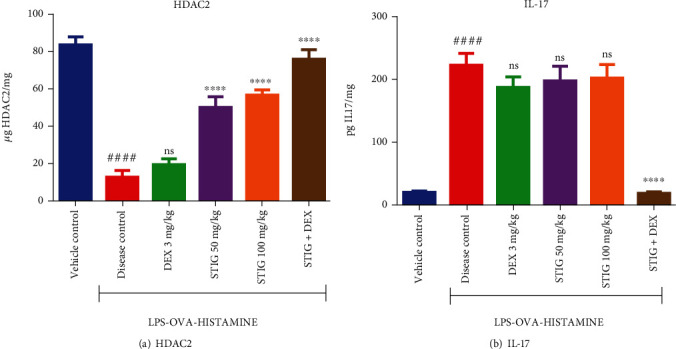
Effect of STIG and STIG+DEX on HDAC2 and IL-17 levels. Data is expressed as mean ± SEM (*n* = 5). ^####^*p* < 0.0001 compared to the vehicle control group; ^∗∗∗∗^*p* < 0.0001, and ns (not significant) as compared to the disease control group with one-way ANOVA followed by Dunnett's *post hoc test.*

Analysis of lung tissue homogenate showed a 7-fold (*p* < 0.0001) reduction in the levels of HDAC2 (to 12.73 ± 3.52 *μ*g/mg protein; Figure 6(a)) and 32-fold (*p* < 0.0001) increase in IL-17 (to 223.60 ± 18.79 pg/mg protein; Figure 6(b)) in the disease control when compared to the vehicle controls (83.54 ± 4.34 *μ*g/mg protein HDAC2 and 7.05 ± 6.85 pg/mg protein IL-17). DEX treatment did not affect downregulation of HDAC2 levels when compared to disease control, but STIG at all the doses tested (50 and 100 mg/kg) caused higher HDAC2 levels (50.08 ± 5.717 and 56.75 ± 2.73 *μ*g/mg respectively) with a 293.40 ± 2.20% (*p* < 0.0001) and 345.78 ± 0.79% (*p* < 0.0001) increase in HDAC2 levels, respectively, when compared to the disease control. As observed for several other analyses in this work, STIG+DEX produced the most effective response, as it caused a 495.83 ± 1.58% (*p* < 0.0001) increase in HDAC2 levels (to 75.85 ± 5.10 *μ*g/mg).

Levels of IL-17 were not significantly affected by neither DEX nor STIG treatment, but STIG+DEX treatment caused a 96.95 ± 12.34% (*p* < 0.0001) reduction in IL-17 levels, at 6.82 ± 6.45 pg/mg IL-17 compared to the disease control (223.60 ± 18.79 pg/mg protein). This effective inhibition of processes that promote airway inflammation once again confirms that combining STIG and DEX may be an effective strategy in managing steroid-resistant asthma.

#### 3.2.2. STIG+DEX Inhibits Smooth Muscle Hyperplasia and Basement Membrane Thickening

**Figure 7:**
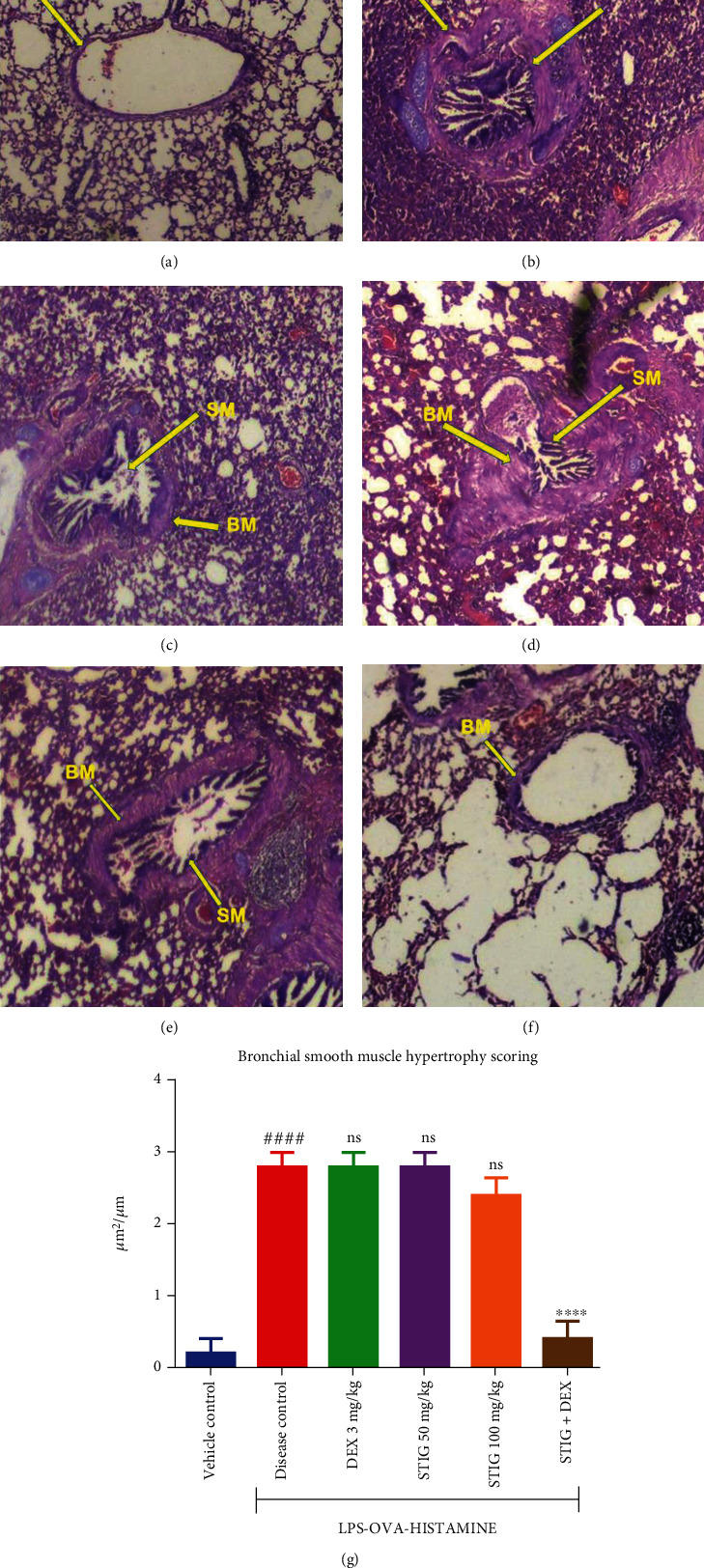
Effect of STIG and STIG+DEX on smooth muscle hypertrophy and basement membrane thickening. Lung tissues were assessed for bronchial basement membrane thickening (BM) and airway smooth muscle hyperplasia (SM) (magnification = ×40). Morphometric analysis quantified the extent of smooth muscle hypertrophy (*g*). Data is expressed as mean index ± SEM (*n* = 5). ^####^*p* < 0.0001 compared to the vehicle control group; ^∗∗∗∗^*p* < 0.0001 and ns (not significant) as compared to the disease control group using ANOVA followed by Dunnett's *post hoc test*.

Disease severity and the effect of the molecular events on the disease were confirmed in histopathological analysis. Associated with steroid-resistant asthma is hypertrophy of smooth muscles of the airway and bronchial basement membrane thickening [25]. Integrity of lungs of the vehicle control group of Guinea pigs (Figure 7(a)) was healthy and normal. The alveolar spaces were patent with no buildup of cells around the bronchioles, the basement membrane was not thickened, and there were no smooth muscles clogging the airway. On the contrary, and as was expected, the disease control group portrayed severe and extensive thickening of the basement membrane with increase smooth muscles enclosing the airway (Figure 7(b)). Interestingly, treatment with DEX and STIG (50 and 100 mg/kg) individually did not reverse these features (Figures 7(c)–7(e), respectively). Nonetheless, STIG+DEX coadministration produced extensive inhibition of bronchial smooth muscle hypertrophy, basement membrane thickening, and bronchial narrowing (Figure 7(f)).

Morphometric analyses using quantitative measurements (0—normal, 1—mild, 2—moderate, and 3—severe; Figure 7(g)) to quantitatively evaluate treatment effects showed that the disease control group had bronchial smooth muscle hypertrophy index (stained area/per unit smooth muscle hypertrophy) of 3.0 ± 0.00 *μ*m^2^/*μ*m compared to 0.2 ± 0.2 *μ*m^2^/*μ*m in the vehicle control, which is a 15-fold (*p* < 0.0001) increase in bronchial smooth muscle hypertrophy due to the disease. Both DEX alone and STIG at 50 mg/kg recorded 2.8 ± 0.20 *μ*m^2^/*μ*m, a 6.77 ± 0.2% decrease in bronchial smooth muscle hypertrophy, while STIG at 100 mg/kg recorded 2.4 ± 0.24 *μ*m^2^/*μ*m, which is a 20.00 ± 0.00% decrease in bronchial smooth muscle hypertrophy. Unsurprisingly, STIG+DEX recorded a bronchial smooth muscle hypertrophy index 0.4 ± 0.24 *μ*m^2^/*μ*m, representing an 86.67 ± 0.24% inhibition. This confirms earlier observations showing that STIG+DEX combination offered the most effective relief in steroid-resistant asthma in Guinea pigs.

## 4. Discussion

Steroid-resistant asthma portrays airway hyperreactivity to allergens, severe inflammatory cell activation and accumulation, airway smooth muscle hypertrophy, submucosal fibrosis, and excessive mucus production resulting in airway remodelling [22, 26]. In steroid-resistant asthma, the presence of LPS in the airway of asthmatics has been considered to contribute to both the worsening of asthma symptoms and resistance to steroids [9, 27]. Previous studies have shown that in the LPS-OVA steroid-resistant asthma model used in this work, the symptoms such as increased mucus secretion, airway inflammation, airway remodelling, bronchoconstriction, and dyspnea are hardly managed by glucocorticoids [9, 10]. Since no effective therapy has been developed to manage steroid-resistant asthma [5, 28], this work sought to test the effectiveness of stigmasterol (STIG), a natural steroid alcohol with potent anti-inflammatory and antioxidant properties in modulating steroid-resistant asthma.

The current work revealed an enormous influx of neutrophils compared to eosinophils into the bronchial airways of the Guinea pigs. Upon treatment, there was significant reduction in neutrophil and eosinophil levels when STIG was used alone and when combined with dexamethasone. This reduction in neutrophil levels is very important in managing steroid-resistant asthma, because, Simpson and colleagues [22] who used clarithromycin in managing steroid-resistant asthma in human subjects showed that, 8 weeks of clarithromycin therapy significantly reduces airway neutrophil numbers and improves quality of life. The significance of reducing neutrophil level was proved by Liao and colleagues [29] as well as Barnes [4], whose works show that in BAL fluid from steroid-resistant asthma patients, neutrophils release enzymes which are destructive to the airway. One of these enzymes is myeloperoxidase (MPO), which enhances the reaction between peroxides and halides to produce hypohalous acids: bleach-like compounds detrimental to the airways [30–32]. Hence, the reduction in both neutrophil and MPO levels by STIG and STIG-DEX combination is mechanistically predictable. Not only does neutrophils produce MPO, they also release malondialdehyde (MDA) which also causes lipid peroxidation and airway inflammation [31, 32]. MDA levels are not increased in airway secretions from patients with mild and moderate asthma [33], but several research works have shown that MDA levels are higher than normal in BAL fluid lavage from patients with steroid-resistant asthma [3, 34, 35]. Increase in MDA levels in steroid-resistant asthma is associated with chronic lipid peroxidation and airway narrowing. In this study, MDA levels increased drastically in the model. However, treatment with STIG caused an appreciable reduction in MDA levels which is in parallel with STIG's efficiency in reducing neutrophil and MPO levels.

Furthermore, substantial correlation between neutrophilia and oxidative stress in steroid-resistant asthma has been identified by several works [36, 37]. Kallapura and colleagues [23] pointed out that neutrophils activate cascade of kinase pathways including mitogen-activated protein kinase pathway which phosphorylates and inactivates nuclear factor erythroid 2-related factor 2 (Nrf2), a crucial enzyme involved in the synthesis of antioxidants such as glutathione, catalase, and superoxide. It is therefore not surprising that the increased level of neutrophils in the disease control group is at par with reduced levels of the antioxidants: glutathione, catalase, and superoxide dismutase indicating that there is oxidative stress. While STIG caused an increase in the level of antioxidants (catalase, glutathione, and superoxide dismutase), a far-reaching increase in antioxidant levels was attained upon combining STIG and DEX. This outcome is consistent with the result of the studies by Zhang and colleagues [32], who showed that increasing antioxidant levels may be effective in managing steroid-resistant asthma since antioxidants are crucial in reducing oxidative stress, a significant factor implicated in steroid resistance as antioxidant levels are found to be low in BAL fluids from steroid-resistant asthma patients [10, 38]. It is therefore possible that the antioxidant effects of STIG may contribute to its efficiency in managing steroid-resistant asthma.

Nitrative stress agents also play an important role in steroid-resistant asthma [24]. The enzyme inducible nitric oxide synthase (iNOS) synthesizes nitric oxide (NO), a compound whose levels are found to increase in exhaled air of steroid-resistant asthma patients [11] which is crucial in the pathogenesis of steroid-resistant asthma. Griendling and colleagues [39] found that the level of this enzyme is increased in lung tissues and bronchoalveolar lavage fluid of steroid-resistant asthma patients. Mechanistic studies by Panda and colleagues [5] demonstrated that nitric oxide produced by inducible nitric oxide synthase (iNOS) causes nitration of cysteine-containing peptides of the glucocorticoid receptor (GR) as well as nitrosylation of its tyrosine moieties resulting in deactivation and degradation of the GR. Deactivation and/or degrading the GR reduces its level, function, and response, a key pathway of steroid resistance in asthma [3, 40]. The levels of NO and the enzyme iNOS were increased upon sensitization and induction of steroid-resistant asthma. The result from this work showed that STIG caused a reduction in iNOS and NO levels when used alone and upon combination with DEX.

An additional factor that contributes substantially to steroid resistance is the reduction in levels of histone deacetylase 2 (HDAC2). According to Peck and Mellins [24], HDAC2 is a proinflammatory gene transcription repressor enzyme which is recruited in glucocorticoid action. Mechanistically, it has been shown that glucocorticoids recruit HDAC2 to perform crucial functions such as deacetylation of histones involved in gene transcription [25, 41, 42]; deacetylation of histones shuts down the transcription of proinflammatory genes. Another function of HDAC2 is to deacetylate the GR; the deacetylation of GR activates it to move from the cytoplasm and bind to glucocorticoid response element in the nucleus for anti-inflammatory gene transcription. Oxidative stress agents on the other hand activate pathways which degrade this useful enzyme [2]. Oxidative stress agents and products such as NO, H_2_O_2_, MDA, and MPO have been documented to individually cause nitration, acetylation, phosphorylation, methylation as well as forming adducts with HDAC2 thereby tagging it for degradation [14, 43]. It was therefore not surprising that dexamethasone is not effective in steroid-resistant asthma as demonstrated with animal models in this work. STIG on the other hand inhibited the degradation of HDAC2, and this effect was even greater with STIG+DEX coadministration. This was not surprising because STIG alone and in combination with DEX reduced oxidative stress in the animals, and this reduction in oxidative stress prevented the damage to HDAC2. This is supported by the work of Trevor and Deshane [2], which demonstrated that reducing oxidative stress agents prevents degradation of HDAC2.

Several research works have indicated increase in levels of IL-17 in lung tissues of deceased subjects of steroid-resistant asthma, and this increase in levels of IL-17 has been associated with activation of pathways which trigger inflammatory responses such as constriction of bronchi, increase capillary permeability, edema, and airway hyperreactivity [10, 42]. IL-17 acts on airway epithelial cells, lung fibroblasts, and other types of inflammatory cells to trigger the production of proinflammatory cytokines, chemokines, and matrix metalloproteinases and promotes the recruitments of neutrophil and macrophage, airway remodelling, and bronchial basement thickening [25, 43]. In this work, STIG alone did not inhibit the elevation of IL-17 significantly; nevertheless, upon combination with DEX, there was a great reduction in IL-17 levels in lung tissues.

To buttress the effect of the treatments on IL-17, airway remodelling and bronchial basement membrane thickening were seen in the lung histology of the disease control group, which revealed bronchial openings filled with smooth muscles, highly thickened basement membrane, and obstruction of bronchioles. In STIG+DEX administration, the airways and bronchioles were patent and the basement membrane was not thickened.

Pharmacological responses from drug combinations as seen in this work are not surprising in drug discovery. The work by Meja and colleagues [34] which combined curcumin and DEX as well as the work of Liao and colleagues [29] which combined andrographolide and DEX to manage COPD found that natural products may enhance the activities of standard drugs or vice versa. This same phenomenon has been seen in this work which shows that combining STIG and DEX may be beneficial in managing steroid-resistant asthma.

## 5. Conclusion

**Figure 8:**
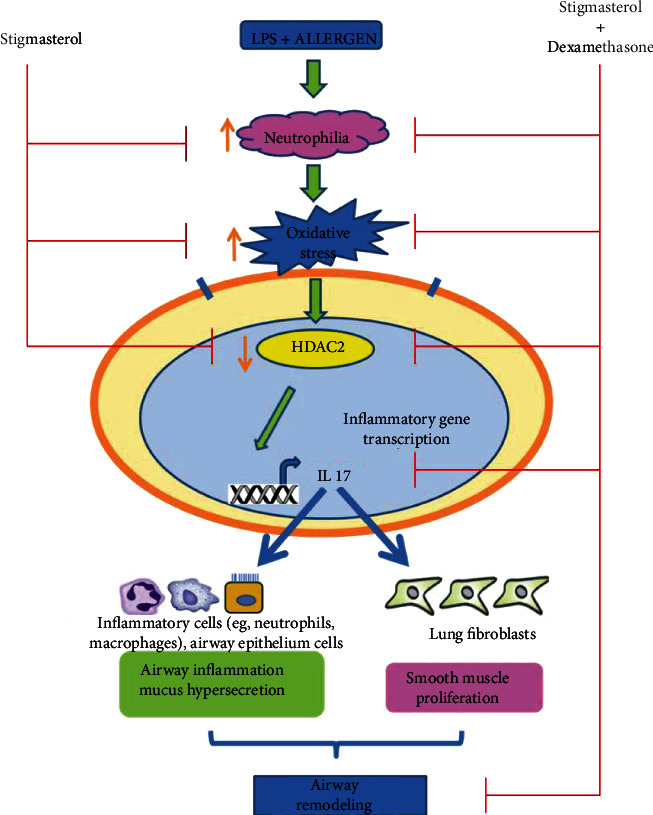
Graphical abstract. Stigmasterol-dexamethasone (STIG+DEX) coadministration is more effective in inhibiting steroid-resistant asthma compared to stigmasterol alone.

Taken together, this work has demonstrated for the first time that, though STIG alone does not modulate steroid-resistant asthma, STIG-DEX combination may be useful in managing steroid-resistant asthma and its associated pathophysiologies (as shown in the graphical abstract, Figure 8).

## Data Availability

The data used to support the findings of this study are available upon request from the corresponding author.

## References

[1] Goleva E., Hauk P. J., Hall C. F. (2008). Corticosteroid-resistant asthma is associated with classical antimicrobial activation of airway macrophages. *The Journal of Allergy and Clinical Immunology*.

[2] Trevor J. L., Deshane J. S. (2014). Refractory asthma: mechanisms, targets, and therapy. *Allergy*.

[3] Adcock I. M., Lane S. J. (2003). Corticosteroid-insensitive asthma: molecular mechanisms. *The Journal of Endocrinology*.

[4] Barnes P. J. (2013). Corticosteroid resistance in patients with asthma and chronic obstructive pulmonary disease. *The Journal of Allergy and Clinical Immunology*.

[5] Panda L., Mabalirajan U. (2018). Recent Updates on Corticosteroid Resistance in Asthma. *European Medical Journal*.

[6] Bucala R. (2012). Approaching the immunophysiology of steroid resistance. *Arthritis Research & Therapy*.

[7] Daan de Boer J., Roelofs J. J., de Vos A. F. (2013). Lipopolysaccharide inhibits Th2 lung inflammation induced by house dust mite allergens in mice. *American Journal of Respiratory Cell and Molecular Biology*.

[8] Lowe A. P., Thomas R. S., Nials A. T., Kidd E. J., Broadley K. J., Ford W. R. (2015). LPS exacerbates functional and inflammatory responses to ovalbumin and decreases sensitivity to inhaled fluticasone propionate in a guinea pig model of asthma. *British Journal of Pharmacology*.

[9] Michel O. (2003). Role of lipopolysaccharide (LPS) in asthma and other pulmonary conditions. *Journal of Endotoxin Research*.

[10] Ray A., Kolls J. K. (2017). Neutrophilic inflammation in asthma and association with disease severity. *Trends in Immunology*.

[11] Toward T. J., Broadley K. J. (2001). Chronic lipopolysaccharide exposure on airway function, cell infiltration, and nitric oxide generation in conscious guinea pigs: effect of rolipram and dexamethasone. *The Journal of Pharmacology and Experimental Therapeutics*.

[12] Ito K., Hanazawa T., Tomita K., Barnes P. J., Adcock I. M. (2004). Oxidative stress reduces histone deacetylase 2 activity and enhances IL-8 gene expression: role of tyrosine nitration. *Biochemical and Biophysical Research Communications*.

[13] Lai T., Tian B., Cao C. (2018). HDAC2 suppresses IL17A-mediated airway remodeling in human and experimental modeling of COPD. *Chest*.

[14] Tomita K., Barnes P. J., Adcock I. M. (2003). The effect of oxidative stress on histone acetylation and IL-8 release. *Biochemical and Biophysical Research Communications*.

[15] Antwi A. O., Obiri D. D., Osafo N. (2017). Stigmasterol modulates allergic airway inflammation in guinea pig model of ovalbumin-induced asthma. *Mediators of Inflammation*.

[16] Walker J. M. (1996). *The Protein Protocols Handbook*.

[17] Heath R. L., Packer L. (1968). Photoperoxidation in isolated chloroplasts. I. Kinetics and stoichiometry of fatty acid peroxidation. *Archives of Biochemistry and Biophysics*.

[18] Sinha A. K. (1972). Colorimetric assay of catalase. *Analytical Biochemistry*.

[19] Misra H. P., Fridovich I. (1972). The role of superoxide anion in the autoxidation of epinephrine and a simple assay for superoxide dismutase. *The Journal of Biological Chemistry*.

[20] Ellman G. L. (1959). Tissue sulfhydryl groups. *Archives of Biochemistry and Biophysics*.

[21] Gibson-Corley K. N., Olivier A. K., Meyerholz D. K. (2013). Principles for valid histopathologic scoring in research. *Veterinary Pathology*.

[22] Simpson J. L., Powell H., Boyle M. J., Scott R. J., Gibson P. G. (2008). Clarithromycin targets neutrophilic airway inflammation in refractory asthma. *American Journal of Respiratory and Critical Care Medicine*.

[23] Kallapura G., Pumford N., Hernandez-Velasco X., Hargisand B. M., Tellez G. (2014). Mechanisms involved in lipopolysaccharide derived ROS and RNS oxidative stress and septic shock. *Journal of Microbiology Research and Reviews*.

[24] Peck A., Mellins E. D. (2009). Breaking old paradigms: Th17 cells in autoimmune arthritis. *Clinical Immunology*.

[25] Schewitz-Bowers L. P., Lait P. J., Copland D. A. (2015). Glucocorticoid-resistant Th17 cells are selectively attenuated by cyclosporine A. *Proceedings of the National Academy of Sciences of the United States of America*.

[26] Antwi A. O., Obiri D. D., Osafo N., Essel L. B., Forkuo A. D., Atobiga C. (2018). Stigmasterol alleviates cutaneous allergic responses in rodents. *BioMed Research International*.

[27] Adesina S., Johnny I., Olayiwola G. (2017). Plants in respiratory disorders I-anti-asthmatics, a review. *British Journal of Pharmaceutical Research*.

[28] Bowler R. P., Crapo J. D. (2002). Oxidative stress in airways: is there a role for extracellular superoxide dismutase?. *American Journal of Respiratory and Critical Care Medicine*.

[29] Liao W., Tan W. S., Wong W. S. (2016). Andrographolide restores steroid sensitivity to block lipopolysaccharide/IFN-gamma-induced IL-27 and airway hyperresponsiveness in mice. *Journal of Immunology*.

[30] Lau D., Mollnau H., Eiserich J. P. (2005). Myeloperoxidase mediates neutrophil activation by association with CD11b/CD18 integrins. *Proceedings of the National Academy of Sciences of the United States of America*.

[31] Wood L. G., Gibson P. G., Garg M. L. (2003). Biomarkers of lipid peroxidation, airway inflammation and asthma. *The European Respiratory Journal*.

[32] Zhang H. X., Liu S. J., Tang X. L. (2016). H2S attenuates LPS-induced acute lung injury by reducing oxidative/nitrative stress and inflammation. *Cellular Physiology and Biochemistry*.

[33] Ito K., Herbert C., Siegle J. S. (2008). Steroid-resistant neutrophilic inflammation in a mouse model of an acute exacerbation of asthma. *American Journal of Respiratory Cell and Molecular Biology*.

[34] Meja K. K., Rajendrasozhan S., Adenuga D. (2008). Curcumin restores corticosteroid function in monocytes exposed to oxidants by maintaining HDAC2. *American Journal of Respiratory Cell and Molecular Biology*.

[35] Tsuchiya K., Siddiqui S., Risse P. A., Hirota N., Martin J. G. (2012). The presence of LPS in OVA inhalations affects airway inflammation and AHR but not remodeling in a rodent model of asthma. *American Journal of Physiology. Lung Cellular and Molecular Physiology*.

[36] Essilfie A. T., Simpson J. L., Dunkley M. L. (2012). Combined Haemophilus influenzae respiratory infection and allergic airways disease drives chronic infection and features of neutrophilic asthma. *Thorax*.

[37] Lee I. T., Shih R. H., Lin C. C., Chen J. T., Yang C. M. (2012). Role of TLR4/NADPH oxidase/ROS-activated p 38 MAPK in VCAM-1 expression induced by lipopolysaccharide in human renal mesangial cells. *Cell Communication and Signaling: CCS*.

[38] Fang S. B., Zhang H. Y., Jiang A. Y. (2018). Human iPSC-MSCs prevent steroid-resistant neutrophilic airway inflammation via modulating Th17 phenotypes. *Stem Cell Research & Therapy*.

[39] Griendling K. K., Touyz R. M., Zweier J. L. (2016). Measurement of reactive oxygen species, reactive nitrogen species, and redox-dependent signaling in the cardiovascular system: a scientific statement from the American Heart Association. *Circulation Research*.

[40] Sartori L., Minucci S. (2015). Tackling oxidative stress by a direct route: a new job for HDAC inhibitors?. *Chemistry & Biology*.

[41] Liu W., Liu S., Verma M. (2017). Mechanism of TH2/TH17-predominant and neutrophilic TH2/TH17-low subtypes of asthma. *The Journal of Allergy and Clinical Immunology*.

[42] Schuliga M. (2015). NF-kappa B signaling in chronic inflammatory airway disease. *Biomolecules*.

[43] Tan W. S. D., Liao W., Zhou S., Wong W. S. F. (2017). Is there a future for andrographolide to be an anti-inflammatory drug? Deciphering its major mechanisms of action. *Biochemical Pharmacology*.

